# The effect of HMGB1 on the clinicopathological and prognostic features of non-small cell lung cancer

**DOI:** 10.18632/oncotarget.7050

**Published:** 2016-01-28

**Authors:** Anlin Feng, Zhenbo Tu, Bingjiao Yin

**Affiliations:** ^1^ Department of Immunology, Tongji Medical College, Huazhong University of Science and Technology, Wuhan, Hubei, People's Republic of China, 430030; ^2^ Department of Immunology, Wuhan University School of Basic Medical Sciences, Wuhan, Hubei, People's Republic of China, 430071

**Keywords:** HMGB1, NSCLC, biomarker, prognosis, ADC

## Abstract

Several studies have assessed the diagnostic and prognostic values of high mobility group protein box 1 (HMGB1) expression in non-small cell lung cancer (NSCLC), but these results remain controversial. The purpose of this study was to perform a meta-analysis of the gene microarray analyses of datasets from the Cancer Genome Atlas (TCGA) and Gene Expression Omnibus (GEO) to evaluate the association of HMGB1 expression with the clinicopathological and prognostic features of patients with NSCLC. Furthermore, we investigated the underlying molecular mechanisms by bioinformatics analysis. Twenty relevant articles involving 2651 patients were included in this meta-analysis; the HMGB1 expression in NSCLC tissues was significantly higher than that in the healthy non-cancer control tissues. We also found an indication by microarray analysis and meta-analysis that HMGB1 expression was associated with the cancer TNM Staging System. In terms of prognostic features, a survival analysis from KM-Plotter tool revealed that the high HMGB1 expression group exhibited poorer survival in lung adenocarcinoma (ADC) and overall NSCLC patients. The survival and disease-free analyses from TCGA datasets also showed that HMGB1 mainly affected the development of patients with ADC. Therefore, we focused on how HMGB1 affected the prognosis and development of ADC using bioinformatics analyses and detected that the mitogen-activated protein kinases (MAPK), apoptosis and cell cycle signaling pathways were the key pathways that varied during HMGB1 up-regulation in ADC. Moreover, various genes such as PLCG2, the phosphatidylinositol-4, 5-bisphosphate 3-kinase superfamily (PI3Ks), protein kinase C (PKC) and DGKZ were selected as hub genes in the gene regulatory network. Our results indicated that HMGB1 is a potential biomarker to predict progression and survival of NSCLC, especially of ADC types.

## INTRODUCTION

HMGB1 is a highly conserved structural transcription factor with a molecular weight of approximately 30 KD. The HMGB1 protein contains three domains: two positively charged DNA-binding motifs (box A and B) and a C-terminal acidic tail. The proximal boxes A and B both contain putative nuclear-emigration signals, while the acidic tail is thought to interact with and protect boxes A and B during emigration from the nucleus [1]. HMGB1 has two main functions depending on the cellular localization. In the nucleus, HMGB1 plays an important role as a DNA-binding protein to sustain nucleosome structure [2] and as an architectural transcription factor regulating gene expression [3, 4]. However, HMGB1 can be released into the extracellular matrix, where it exerts crucial functions in inflammation and carcinogenesis through its receptors including receptor for advanced glycation end-products (RAGE), the toll-like receptor (TLR) 2, and TLR 4 [5–7]. Recent studies have shown that the HMGB1 gene is highly expressed in various cancers, showing oncogene-like biomarkers of these cancers [8–11]. Extracellular HMGB1 has multiple pro-tumor roles in tumorigenesis, such as promotion of angiogenesis, evasion of apoptosis, inhibition of antitumor immunity, inflammation, promotion of tissue invasion and metastasis [12–16].

Lung cancer is the leading cause of cancer-related mortality in both sexes. It has been traditionally subdivided into two principal groups, named small cell lung cancer and non-small cell lung cancer (NSCLC); the latter type consists of 85%-90% of lung cancer diagnoses. NSCLC mainly includes adenocarcinoma (ADC) and squamous cell carcinoma (SCC) as histologic types. Despite diverse treatment methods such as surgery, chemotherapy, radiation and targeted therapies, the overall 5-year survival rate for NSCLC is only 18.2% [17]. The high mortality rates of NSCLC are partially due to the lack of effective prognostic factors such as biomarkers. The clinical behavior of NSCLC is mainly dependent on its stage; there are still many difficulties in significantly improving survival of NSCLC because lung cancer masses of patients are diagnosed at advanced stages with local or distant metastasis. Therefore, identifying novel prognostic factors as biomarkers may be a clinically useful tool for early detection of NSCLC.

Many studies have examined the relationship between HMGB1 expression and survival in patients with NSCLC. However, the diagnostic and prognostic value of HMGB1 for NSCLC has yet to be confirmed. Therefore, we performed meta-analysis and Gene Expression Omnibus (GEO) dataset parameters to disclose the association between the expressions of HMGB1 and clinicopathological or prognostic factors of NSCLC. Having found that HMGB1 expression mainly effects the survival rates of ADC by analysis of The Cancer Genome Atlas (TCGA) and KM-Plotter tool, we then used functional and network analysis to detect important signaling pathways as well as to detect key genes in order to better understand the mechanisms of HMGB1 contributing to the development of ADC.

## RESULTS

### HMGB1 is up-regulated in NSCLC patients

**Figure 1:**
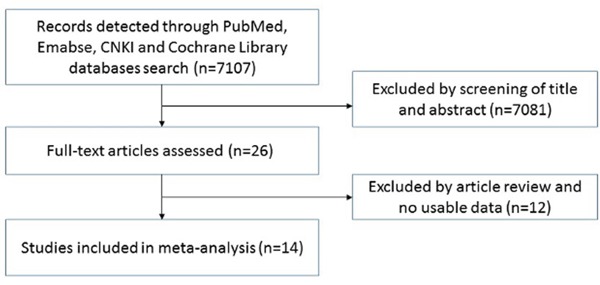
Schematic flow diagram of article selection

Twenty-six articles were assessed by titles or abstracts in PubMed, Embase, Cochrane Library and CNKI databases (see Figure 1). After the main texts were checked carefully, 14 articles with 2048 cases met the standard of this research. These studies mainly concentrated on the expression of HMGB1 with clinicopathological characteristics or prognostic factors for NSCLC. We collected the following items from every study: first author, year of publication, country, number of patients, and detection methods (Table 1). Six microarray datasets (GSE19188, GSE21933, GSE30219, GSE40275, GSE51855 and GSE56044), which included both NSCLC patients and healthy people, were also collected from the GEO and ArrayExpress databases until September 2015 (Table 1).

**Table 1:** Main characteristics of included studies in the meta-analysis

Author	Year	Country	Duration Months	Patients Number	Detection Methods
Arima C[18]	2014	Japan	91	126	cDNA Microarray
Cui ZS[19]	2014	China	5	30	Elisa
He DP[20]	2014	China	24	48	RT-PCR, IHC
He JJ[21]	2012	China	40	65	IHC
Hou J[22]	2010	Netherlands	NR*	82	cDNA Microarray
Karlsson A[23]	2014	Sweden	NR*	121	cDNA Microarray
Kastner S	2012	Austria	NR*	16	cDNA Microarray
Lo FY[24]	2012	Taiwan, China	NR*	21	cDNA Microarray
Naumnik W[25]	2009	Poland	NR*	40	Elisa
Rousseaux S[26]	2013	France	NR*	272	cDNA Microarray
Shang GH[27]	2009	China	42	145	Elisa
Shen X[28]	2009	China	24	63	RT-PCR, WB
Su WM[29]	2012	China	24	69	IHC
Wang FL[30]	2009	China	7	53	IHC
Wang JY[31]	2014	China	3	30	Elisa, IHC
Wang Y[32]	2014	China	12	30	RT-PCR, Elisa
Xu SB[33]	2009	China	48	52	IHC
Yang XM[34]	2013	China	24	64	RT-PCR
Zhang SD[35]	2011	China	24	95	IHC
Zhang X[36]	2013	China	36	106	IHC

* NR: not reporting.

**Figure 2:**
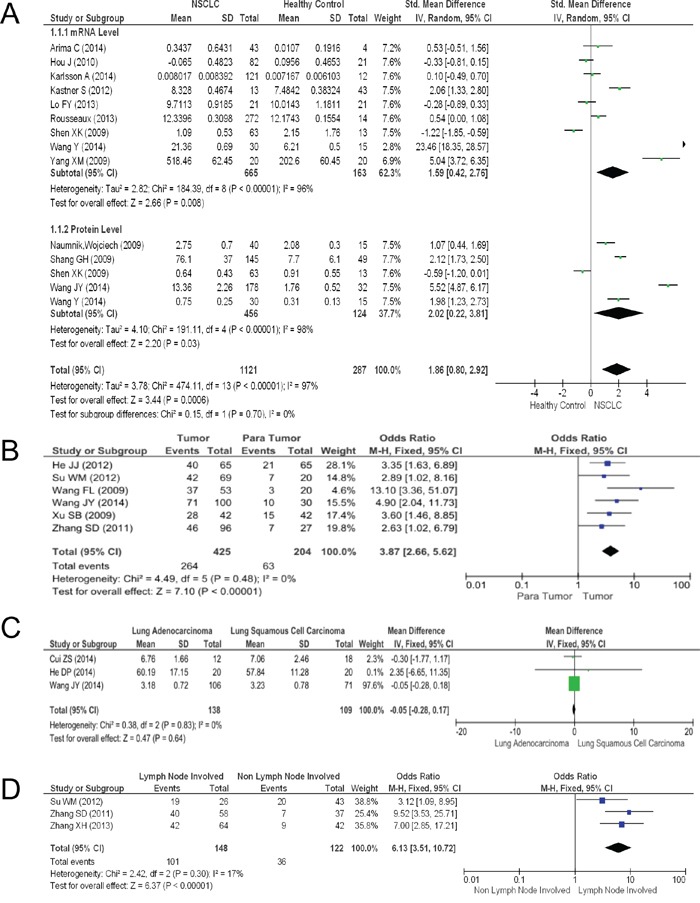
The results of the meta-analysis **A.** Forest plot for HMGB1 in NSCLC and normal lung tissues. **B.** Forest plot for HMGB1 in NSCLC and para-tumor tissues. **C.** Forest plot for HMGB1 in lung ADC and SCC. **D.** Association between HMGB1 expression and NSCLC lymph node metastasis.

The expression levels of HMGB1 in NSCLC tissues were significantly higher than in healthy non-cancer control tissues. The pooled mean difference for NSCLC versus normal people in mRNA was 1.59 (9 studies, 764 patients, 95% CI 0.42-2.76, Z=2.66, P=0.000, see Figure 2A), while that for the protein level was 2.02 (6 studies, 741 patients, 95% CI 0.22-3.81, Z=2.2, P=0.000, see Figure 2A). The pooled model also showed a significantly higher protein level of HMGB1 in NSCLC tissues than in para-tumor tissues using IHC methods (6 articles, pooled mean difference 3.87, 95% CI 2.66-5.62, Z=7.1, P=0.000, see Figure 2B). A Begg's funnel plot was performed to detect the potential publication bias of the above studies. The funnel plots were generally symmetric (Supplementary Figure S1A-B), indicating the absence of publication bias of our results.

### Relationship between HMGB1 and clinicopathological features of NSCLC

We compared HMGB1 expression levels in two main histologic types (ADC and SCC) and metastasis (non-lymph node and lymph node metastasis, see Figure 2). The contrast between these two main histologic types had no statistical significance (3 articles, pooled mean difference −0.05, 95% CI −0.28-0.17, Z=0.47, P=0.64, see Figure 2C). However, a statistical significance in the lymph node metastasis comparison between the two histologic types was observed (3 articles, pooled mean difference 6.13, 95% CI 3.51-10.72, Z=6.37, P=0.000, see Figure 2D). Nevertheless, no obvious publication bias was found in funnel plots.

**Figure 3:**
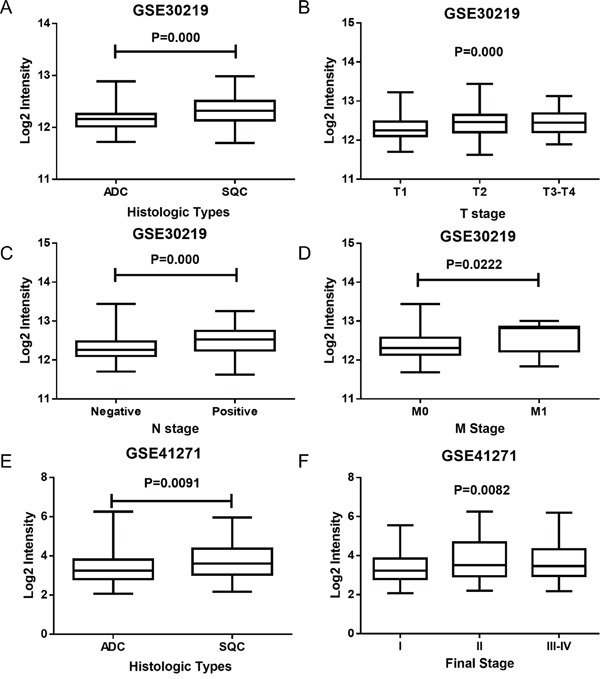
The expression of HMGB1 in various histologic types and TNM stages in NSCLC **A.** The expression of HMGB1 in ADC and SCC (GSE30219). **B-D.** The expression of HMGB1 in different TNM stages (GSE30219). **E.** The expression of HMGB1 in ADC and SCC (GSE41271). **F.** The expression of HMGB1 in different final stages (GSE41271).

Whole genomic expression profiles detected by gene chip technology supplied unbiased quantitative measures of mRNA levels, and it could be used to calculate the relationship between histopathological characteristics and the target gene. Upon examining the HMGB1 expression in NSCLC patients in GSE30219 and GSE41271, we found some intriguing results. In GSE30219, HMGB1 expression in ADC and SCC was found to be significantly different (Figure 3A, P=0.000); it was also found to be significantly different in tumor size (T stage, P=0.000), lymph node involvement (N stage P=0.000) and distant metastasis (M stage P=0.022) (Figure 3B–3D). In GSE41271, HMGB1 was observed to have a significant difference in final stage (P=0.0082) and histologic types (P=0.0091) (Figure 3E–3F).

Having examined HMGB1 expression in various histologic types and TNM stages from GSE30219 and GSE41271, we then associated the expression level of HMGB1 (low and high expressing groups) with clinicopathological parameters. It can be shown from the results that there was an increase in the percentage of SCC subclass patients (54.10%) expressing higher levels of HMGB1 than ADC (20.00%). In addition, it was observed that patients in T Stage II (66.67%) and III-IV (61.54%) had an increased level of HMGB1 expression compared to patients in T Stage I (44.58%), and an increased percentage of patients with lymph node involvement (N Stage Positive, P=68.82%) showed increased HMGB1 expression (see Table 2). Similarly, GSE41271 also showed an increase in the percentage of SCC subclass patients (60.49%), showing increased levels of HMGB1 greater than ADC (43.55%), and the final stage II (58.00%) and III-IV (57.61%) also indicated an increased percentage of patients with HMGB1 high expression compared to final stage I patients (42.11%) (see Table 3). Furthermore, no major relationships were found between HMGB1 low and high expression and the other clinicopathological parameters (age, sex and M stage smoking) in both GSE30219 and GSE41271 datasets.

**Table 2:** HMGB1 levels and clinicopathological parameters in patients with NSCLC (GSE30219)

Pathology Character	n	HMGB1 Expression		P Value
		Low	High	
**Age(year)**				0.369
<=60	124	65	59	
>60	147	69	78	
**Sex**				0.338
Female	43	23	20	
Male	250	114	136	
**Histology**				0.000*
ADC	85	68	17	
SCC	61	28	33	
**T Stage**				0.003*
IA-IB	166	92	74	
IIA-IIB	69	23	46	
IIIA-IIIB, IV	52	20	32	
**N Stage**				0.000*
Negative	198	107	91	
Positive	93	29	64	
**Metastasis**				0.290^#^
No	282	134	148	
Yes	8	2	6	

* P<0.05

# Calculated with Fisher's exact test.

**Table 3:** HMGB1 levels and clinicopathological parameters in patients with NSCLC (GSE41271)

Pathology Character	n	HMGB1 Expression		P Value
		Low	High	
**Age(year)**				0.583
<=60	98	51	47	
>60	177	86	91	
**Sex**				0.302
Female	127	59	68	
Male	148	78	70	
**Smoke**				0.311
Non	27	16	11	
Yes	247	121	126	
**Histology**				0.011*
ADC	186	105	81	
SCC	81	32	49	
**Final Stage**				0.035*
IA-IB	133	77	56	
IIA-IIB	50	21	29	
IIIA-IIIB, IV	92	39	53	

* P<0.05

### HMGB1 expression was related to the survival rate of NSCLC patients

**Figure 4:**
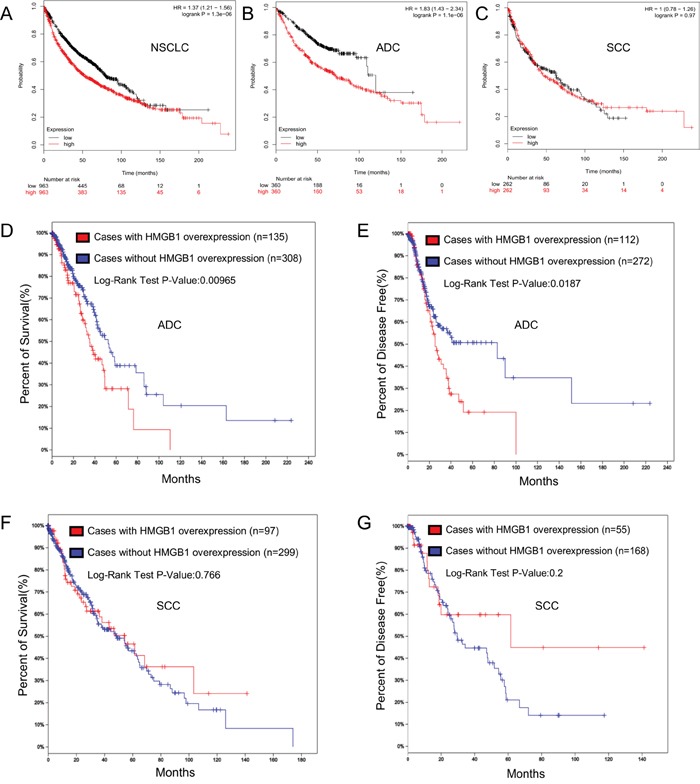
HMGB1 expression is correlated with the survival rate of NSCLC patients **A.** Overall survival rate was analyzed in 1928 NSCLC patients in relation to HMGB1 expression. **B.** Overall survival rate was analyzed in 866 ADC patients in relation to HMGB1 expression. **C.** Overall survival rate was analyzed in 675 SCC patients in relation to HMGB1 expression. **D-E.** Overall survival and disease-free time rate were analyzed in 576 lung ADC patients in relation to HMGB1 expression. **F-G.** Overall survival and disease-free time rate were analyzed in 504 cases of lung SCC.

We further performed a survival analysis to detect the survival differences for all NSCLC patients in 14 NSCLC Affymetrix microarray datasets with 1928 samples using the online KM-Potter tool [37]. Log-rank and Kaplan-Meier tests were used to compare the survival of patients, and the results indicated that survival time was significantly dissimilar between high and low expression groups in NSCLC (P=0.000) (Figure 4A). The differences between the two main histologic types in NSCLC were also considered; we additionally performed survival analysis on ADC and SCC patients in all datasets and only found significant differences in ADC (P=0.000) (Figure 4B–4C).

TCGA (http://cancergenome.nih.gov/) is a project to catalogue genetic mutations responsible for cancer by bioinformatics methods and genome sequencing, while the cBioPortal [38, 39] (http://www.cbioportal.org/index.do) is an exploratory analysis tool for exploring datasets from TCGA to find pathways of interest in one or more cancer types. The TCGA data (Lung Adenocarcinoma, Provisional) was analyzed by cBioPortal, and it showed that HMGB1 was over-expressed in 26% of cases (Supplementary Figure S2A). The HMGB1 over-expression was significantly associated with a poorer overall survival rate and shorter disease-free time (Figure 4D–4E). After that, we scrutinized the Lung Squamous Cell Carcinoma (TCGA, Provisional) dataset for changes in expression of HMGB1 and observed 148 cases in which HMGB1 was over-expressed out of 504 cases (24%) (Supplementary Figure S2B). However, the HMGB1 over-expression did not show statistical significance with the overall survival rate and disease-free time (Figure 4F–4G). No mutation of HMGB1 was found in these two TCGA datasets.

### The molecular mechanisms of HMGB1 in NSCLC

Our results noted that HMGB1 should mostly affect the survival and prognosis of lung adenocarcinoma; however, the molecular mechanisms at play were not so clear. Therefore, we used bioinformatics analysis techniques to analyze the pathway and gene information in GSE30219 during HMGB1 up-regulation. To explore the transcriptional profile determined by HMGB1 expression, we compared the high and low HMGB1 expression groups. In ADC, 1672 genes were up-regulated and 1184 genes (see Supplementary Table 1) were down-regulated in the high HMGB1 expression group. In 54675 detected probes, 5.22% of the genes were differentially expressed.

**Figure 5:**
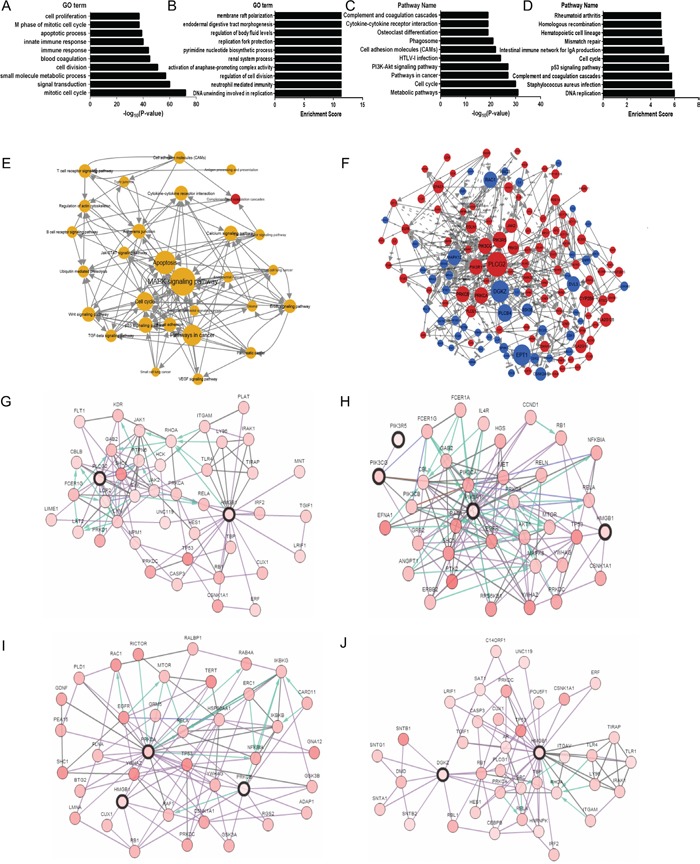
The bioinformatics analysis of molecular mechanisms of HMGB1 in ADC **A-B.** Significantly over-represented biological processes in DEGs. **C-D.** Significantly over-represented pathways in DEGs. **E.** Pathway network. The dots represent pathways; the size of the dots represents the value of the degree of expression; the red and blue color of the dots denote up-regulated and down-regulated pathways, respectively; the yellow color of dots indicates that this pathway contains both up-regulated and down-regulated genes; and the direction of the arrow indicates upstream and downstream. **F.** Gene regulatory network. The dots represent genes; the size of the dots represents the value of betweenness centrality; the red and blue color of the dots denote up-regulated and down-regulated genes, respectively; the direction of the arrow denotes the upstream and downstream relationship; and the dotted lines denote indirect interaction. **G.** Network view of the PLCG2/HMGB1 neighborhood, PLCG2 and HMGB1 are seed genes (indicated with thick border). Darker red indicates increased frequency of alteration in ADC. **H.** Network view of the PIK3CG/PIK3R5/PIK3R1/HMGB1 neighborhood, PIK3CG, PIK3R5, PIK3R1 and HMGB1 are seed genes (indicated with thick border). **I.** Network Figure S view of the PRKCA/PRKCB/HMGB1 neighborhood, PRKCA, PRKCB and HMGB1 are seed genes (indicated with thick border). **J.** Network view of the DGKZ/HMGB1 neighborhood, DGKZ and HMGB1 are seed genes (indicated with thick border).

Gene Ontology (GO) is a major gene-set database to control the vocabulary of the biological process. The result of GO analysis toward DEGs (both upregulated and downregulated genes) showed that the genes were significantly enriched for P-value in several GO terms, such as signal transduction, small molecule metabolic process, transcription, immune response, and apoptotic process. These biological processes had the highest enrichment scores that was involved in DNA replication, neutrophil-mediated immunity, regulation of cell division, etc. (Figure 5A–5B). Kyoto Encyclopedia of Genes and Genomes (KEGG) pathway analysis can find significant pathways in which DEGs (both upregulated and downregulated genes) participate. The mostly significant pathways for P-values from the KEGG pathway database included Metabolic, PI3K-Akt, cell cycle, pathways in cancer and others, and the pathways that possessed more enrichment scores were DNA replication, Staphylococcus aureus infection, complement and coagulation cascades and p53 signaling pathway. (Figure 5C–5D).

The degree within the pathway network, which describes the count of single pathway that regulates other pathways, is represented by the size of the dots. The pathway appears more central within the network if it possesses a higher degree. Pathway network analysis showed that MAPK, apoptosis, p53, JAK-STAT and calcium signaling pathways possessed a higher degree in all altered signaling pathways (Figure 5E). Furthermore, we wanted to find the hub genes within the gene regulatory networks, which were built by KEGG pathways. Additionally, the main central genes were determined by betweenness values, which are an indicator of a node's centrality within a network in which a node with a high betweenness value possesses a large influence on the effect and transfer of other genes within the network. We observed that PLCG2, PI3Ks genes (PIK3CG, PIK3R5, PIK3R1), PKC genes (PRKCA, PRKCB), and DGKZ were the main central genes based on betweenness values (Figure 5F). Using cBioPortal, we drew four networks, which showed the interactions between HMGB1 and other key genes (see Figure 5G-5J), and only PRKCA was found to directly interact with HMGB1 (Figure 5I), and PIK3R1and PRKCB could be linked to HMGB1 by RB1 (see Figure 5H, 5I).

Lastly, our bioinformatics results show that several biological processes (mitosis process, mitotic cell cycle, cell division, DNA repair, etc., see Supplementary Figure S3A-B) and pathways (cell cycle, DNA replication and metabolic pathways, etc., see Supplementary Figure S3C-D) are the most influenced hub items from GO/KEGG during HMGB1 up-regulation in SCC. Pathway network analysis showed that ubiquitin mediated proteolysis, pathways in cancer and cell cycle possessed a higher degree in all altered signaling pathways (see Supplementary Figure S3E) in SCC. However, the most influenced and key processes such as MAPK signaling pathway, apoptosis and PI3K-Akt during HMGB1 up-regulation in ADC may not play a significant role in SCC.

## DISCUSSION

Several studies report that HMGB1 plays a key role in various types of malignancies, such as breast cancer [40], gastric cancer [41] and hepatocellular carcinoma [42]. However, certain factors remain unclear that are related to the overexpression and prognosis of HMGB1 in NSCLC. Here, our results show that the expression of HMGB1 is higher in NSCLC tissues than healthy non-cancer control tissues, leading to poor prognosis and correlating with cancer TNM stages. The overexpression of HMGB1 has a variety of functions in the progression of ADC, including metabolic process, apoptosis, cell proliferation and metastasis.

According to our results and expectations, HMGB1 was over-expressed in NSCLC tissues, and some of the reasons that may lead to elevation of HMGB1 in serum and tissues of NSCLC patients might include the secretion of HMGB1 by lung cancer cells and that the expression of HMGB1 increased in primary ADC tissues compared to non-cancerous tissues [43]. Other reasons might involve the secretion of HMGB1 from apoptotic or necrotic cells from the core region in lung cancer tissues. We also considered that the mutation of HMGB1 might lead to up-regulation in lung cancer, but the results from TCGA exclude this possibility. We found three studies that showed higher expression of HMGB1 was detected in advanced stage NSCLC patients [27, 28, 44]. Similar results from Liu PL [44] showed that NSCLC patients with a high level of HMGB1 were associated with a poor clinical prognosis, but this article was limited by the number of cases (n=48). Chang YH [45] found that a 21-gene signature in the HMGB1/RAGE signaling pathway was significantly associated with prognosis of ADC, which agrees with our survival analysis results from GEO and TCGA datasets. This agreement therefore suggests that the up-regulation of HMGB1 in ADC can predict a poorer prognosis.

Some of the mechanisms through which HMGB1 promotes the development of cancer could include the following: first, HMGB1 and RAGE coordinately boosted tumor cell mitochondrial complex I activity, adenosine triphosphate (ATP) synthesis and tumor cell proliferation to increase the requirements of tumor cells [15, 16]. Second, extracellular HMGB1 induced caspase-1 activation through RAGE and TLR4 pathways to increase the expression of multiple inflammatory mediators, which could promote metastasis and invasion of cancer cells [12, 13] and lastly, HMGB1 was also involved in endothelium cell (EC) activation and angiogenic activity through the MAPK, ERK and JNK pathways, acting as a proangiogenic cytokine [14, 46]. The pathway network analysis showed that the MAPK signaling pathway and apoptosis were located at the center of differentiated pathways (Figure 5C). As a downstream signaling pathway activated by extracellular HMGB1, MAPK pathways are related to the progression of several cancers and play an important role in cancer cell growth and malignant transformation [47, 48]. HMGB1 might relate to the evasion of apoptosis in lung ADC. Other key pathways such as p53, the cell cycle, JAK-STAT and calcium signaling pathways were found to be involved in the development of several cancers (Figure 5C). For the hub genes in our regulating network, PLCG2 possesses the highest nodes in all DEGs and encodes the phospholipase cγ2 (PLC γ2) enzyme, which regulates cell proliferation and apoptosis [49, 50]. Three genes (PIK3CG, PIK3R5 and PIK3R1) encoding class I PI3Ks have been linked to cellular functions such as cell growth, survival, proliferation and migration. The PKC related genes (PPRKCA and PRKCB) mainly function in regulating cell proliferation [51]. In addition, the down-regulated hub gene DGKZ encodes DGKZ, which is known to attenuate PKC activity. The pathway-related networks provide us with a number of potential genes or pathways that may relate to metabolic, metastasis and angiogenesis functions of HMGB1 in ADC; hence, the call for further investigations.

We wonder what the differences are between ADC and SCC that could cause these different prognostic results. To date, ADC is the most diagnosed histologic subtype of NSCLC, followed by SCC [52]. ADC's mutational landscape is different from that of SCC [53], which leads to the different pathways alternations. ADC is focused on receptor tyrosine kinase (RTK)/Ras/MAPK, PI3K/AKT/mTOR and JAK-STAT pathways, as has been shown here by bioinformatics analysis, while SCC etiology is mainly concentrated on cell cycles, DNA repair and oxidative stress response. Mutations in RTK signaling are more frequent in ADC [54, 55]. Therefore, we hypothesized that some pathways, such as the MAPK signaling pathway, were more likely altered signaling pathways and thus played a key role in the progression of ADC during HMGB1 up-regulation compared with SCC.

Some of the limitations of our study included the following: first, different researchers used different detection methods to assess HMGB1 expression in the included articles; thus, there was possible heterogeneity. Second, publication bias could not be totally dismissed because negative results were not as conclusive as the positive results. Third, the number of included articles was limited, as has been shown by our literature search. Finally, the molecular functions of HMGB1 towards ADC were analyzed mostly by bioinformatics analysis. Therefore, the biological role of HMGB and its hub genes in tumorigenesis should be investigated in further experimental studies.

In summary, we found that HMGB1 levels were elevated in NSCLC tissues over those of healthy non-cancer control tissues and were closely related to histologic types and TNM stage; however, HMGB1 only affected the survival time of lung cancer subclass (ADC) patients. Then, we performed a comprehensive bioinformatics analysis of DEGs and revealed possible central signal pathways (MAPK, apoptosis and cell cycle) and genes (PLCG2, PI3Ks, PKC and DGKZ) in the development of lung ADC. Therefore, this could aid the understanding of HMGB1 effects in NSCLC and reveal potential targets for diagnostic and therapeutic manipulation.

## MATERIALS AND METHODS

### Meta-analysis

We searched these databases without any language restrictions: Cochrane Library (1974-2015), PubMed (1966-2015), Embase (1974-2015) and CNKI (2000-2015) databases, using the following keywords for the literature search: (“HMGB1” OR “high motility group box 1”) AND (“NSCLC” OR “Non-small cell lung cancer” OR “Non-small cell lung carcinoma”). The inclusion criteria included: 1) the articles that evaluated the relationship between HMGB1 expression and the clinicopathological significance and prognostic factors of NSCLC; 2) serum HMGB1 expression was measured by enzyme-linked immunosorbent assay (ELISA); and 3) tissue HMGB1 expression was measured by real-time reverse transcription polymerase chain reaction (RT-PCR), immunohistochemistry (IHC) or western blot (WB). We also collected NSCLC mRNA microarray datasets from GEO Datasets (http://www.ncbi.nlm.nih.gov/gds). The following keywords were used in our search: (“lung cancer”) AND (“Homo sapiens”). Eligible datasets were included if they met the following criteria: 1) both NSCLC patients and healthy people were included in each dataset, which contained more than 10 samples; 2) patients did not receive any treatment; and 3) the expression data of HMGB1 from the patient and control groups were not provided or could be calculated. The Series Matrix Files of microarray datasets, which has already been normalized and background corrected, were directly downloaded from the GEO web site. Two reviewers (Anlin Feng and Zhenbo Tu) extracted all data into a standardized data form that included the first author's name, publication year, study population and region, number of cases and controls, stage of lung cancer, and HMGB1 expression. We performed a meta-analysis using the Review Manager 5.3 program. The heterogeneity analysis among studies was assessed using the I2 index and Q-test (P<0.05). The mean differences with 95% confidence intervals estimates were calculated by random-effect models or fixed-effect models (a random effect model for P<0.1 and I>50%; a fixed effect model for P>0.1 and I<50%). Potential publication bias was measured by funnel plots.

### Clinical parameters and HMGB1 expression

In order to further investigate the prognostic impact of HMGB1 mRNA on NSCLC, lung cancer gene expression data and the corresponding clinical data used in this study were obtained from the publicly available GEO database. Finally, the GSE30219, GSE41271 datasets were chose because of the large number of NSCLC patient samples and correspondingly complete clinical information. We divided samples into two groups in accordance with the median value of expression of HMGB1. The χ2 test or Fisher's exact test were performed to clarify the relationship between HMGB1 expression and clinical parameters. Statistical analyses were conducted using GraphPad Prism 6 software, and P<0.05 was considered statistically significant.

Survival differences were validated at the gene expression level by KM-Plotter (http://www.kmplot.com/analysis/index.php?p=service&cancer=lung). KM-Plotter tool was an online survival analysis software to assess the prognostic value of biomarkers using transcriptomic data in various cancers and contained 1928 NSCLC patients with survival data. HMGB1 was entered as the gene symbol, and the median value of HMGB1 expression was selected as the cut-off of the high and low HMGB1 groups. Univariate Cox regression was performed to compute the HR and P values. The Kaplan-Meier and Log-Rank tests were used to estimate and display the outcomes.

### cBioPortal cancer genomic data sets analysis

The other web application, cBioPortal, was used to analyze the survival rate in HMGB1 high and HMGB1 low groups. We chose the two largest datasets up to now, with each having more than 500 cases: Lung Adenocarcinoma (TCGA, Provisional) (http://www.cbioportal.org/study.do?cancer_study_id=luad_tcga#dc-plots) and Lung Squamous Cell Carcinoma (TCGA, Provisional) (http://www.cbioportal.org/study.do?cancer_study_id=lusc_tcga) from the TCGA database. We defined genetic alterations as HMGB1 mRNA over-expression if greater than the mean value. For the genomic profiles, we selected these four options: mutations, putative copy-number alterations from GISTIC, mRNA expression data and protein/phosphoprotein level.

### Bioinformatics analysis

We mainly used the web site of the Gene-Cloud of Biotechnology Information (GCBI, https://www.gcbi.com.cn/gclib/html/index) to explore information about pathways and genes from DEGs between high and low HMGB1 expression groups in lung adenocarcinoma and squamous cell carcinoma. Affymetrix microarray dataset GSE30219 with the largest sample size within all NSCLC GEO datasets was selected to perform bioinformatics analysis. Before analyzing data for biological variation, GCBI performed data processing (which included data normalization and filtering of flagged data) and quality control. GCBI used the robust multi-chip average algorithm to calculate the expression level of probes. The DEGs were screened from the high and low HMGB1 expression groups and were exhibited as volcano plots using 1.2 for differential multiples and 0.05 for P values in every contrast as cut-off values.

GO analysis (http://geneontology.org/) was performed on the DEGs. For each contrast, we detected enriched categories, applied a hypergeometric test on the gene lists against the universe of all the expressed genes, and then selected the most significant GO sets related to the expression level of HMGB1, which had both the P value less than 0.05 and false discovery rate less than 0.05. We used Fisher's exact test for DEGs and KEGG (http://www.genome.jp/kegg/pathway.html) to find the obvious altered pathways (P value <0.05) during which HMGB1 increased. Based on the interaction relationship from KEGG, we built pathways and gene regulatory networks for GCBI.

## SUPPLEMENTARY FIGURES AND TABLE




