# Supplementary material for: The effect of HMGB1 on the clinicopathological and prognostic features of non-small cell lung cancer
Source: Oncotarget. 2016 Jan 28;7(15):20507–19. doi: 10.18632/oncotarget.7050 (PMC4991471; doi:10.18632/oncotarget.7050)
Supplement: Supplementary file 1 [file oncotarget-07-20507-s001.pdf]

SUPPLEMENTARY FIGURES AND TABLE

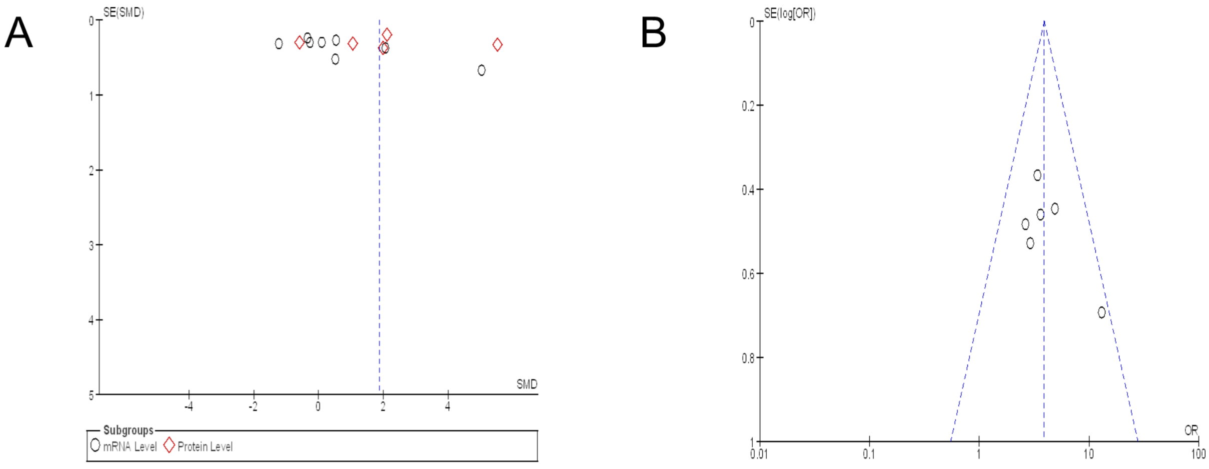

Supplementary Figure S1: Funnel plot for meta-analysis. A. Funnel plot for HMGB1 in NSCLC and normal tissues. **B.** Funnel plot for HMGB1 in tumor and para-tumor tissues.

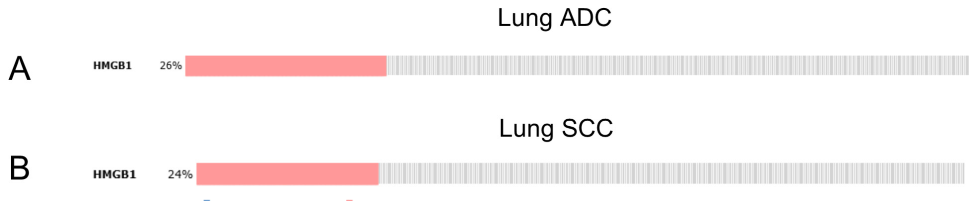

Supplementary Figure S2: The results of analysis for TCGA datasets. A. HMGB1 was altered in 148 (28%) of 576 cases in lung adenocarcinoma (TCGA, Provisional). **B.** HMGB1 was altered in 119 (24%) of 504 cases in lung squamous cell carcinoma (TCGA, Provisional).

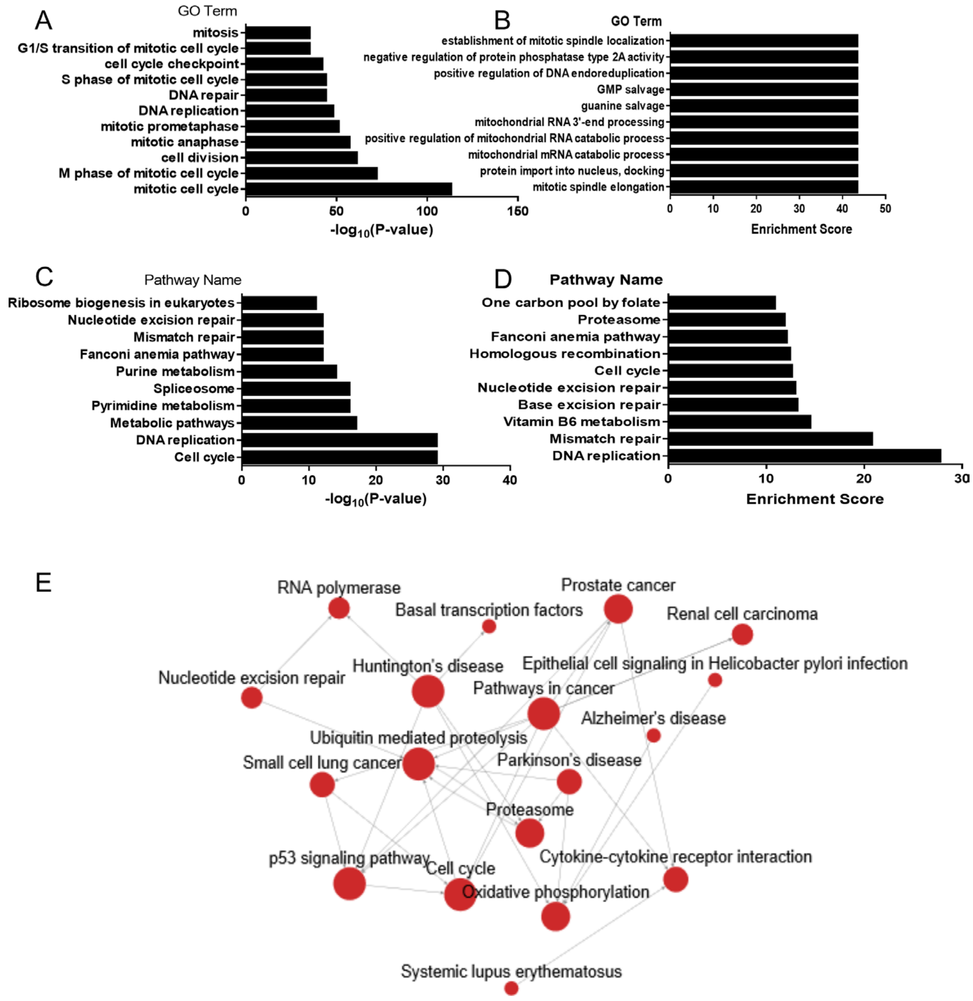

Supplementary Figure S3: The bioinformatics analysis of molecular mechanisms of HMGB1 in SCC. A-B. Significantly over-represented biological processes in DEGs. **C-D.** Significantly over-represented pathways in DEGs. **E.** Pathway network.

Supplementary Table S1

See Supplementary File 1
